# Understanding security tactics in microservice APIs using annotated software architecture decomposition models – a controlled experiment

**DOI:** 10.1007/s10664-024-10601-1

**Published:** 2025-02-14

**Authors:** Patric Genfer, Souhaila Serbout, Georg Simhandl, Uwe Zdun, Cesare Pautasso

**Affiliations:** 1 Research Group Software Architecture, Faculty of Computer Science and UniVie Doctoral School Computer Science DoCS, University of Vienna, Vienna, Austria; 2 Software Institute (USI), Lugano, Switzerland; 3 Research Group Software Architecture, Faculty of Computer Science, University of Vienna, Vienna, Austria

**Keywords:** Microservice architecture, Microservice security, Software architecture metrics, Component diagrams, Controlled experiment, Empirical software engineering, Understandability

## Abstract

While microservice architectures have become a widespread option for designing distributed applications, designing secure microservice systems remains challenging. Although various security-related guidelines and practices exist, these systems’ sheer size, complex communication structures, and polyglot tech stacks make it difficult to manually validate whether adequate security tactics are applied throughout their architecture. To address these challenges, we have devised a novel solution that involves the automatic generation of security-annotated software decomposition models and the utilization of security-based metrics to guide software architectures through the assessment of security tactics employed within microservice systems. To evaluate the effectiveness of our artifacts, we conducted a controlled experiment where we asked 60 students from two universities and ten experts from the industry to identify and assess the security features of two microservice reference systems. During the experiment, we tracked the correctness of their answers and the time they needed to solve the given tasks to measure how well they could understand the security tactics applied in the reference systems. Our results indicate that the supplemental material significantly improved the correctness of the participants’ answers without requiring them to consult the documentation more. Most participants also stated in a self-assessment that their understanding of the security tactics used in the systems improved significantly because of the provided material, with the additional diagrams considered very helpful. In contrast, the perception of architectural metrics varied widely. We could also show that novice developers benefited most from the supplementary diagrams. In contrast, senior developers could rely on their experience to compensate for the lack of additional help. Contrary to our expectations, we found no significant correlation between the time spent solving the tasks and the overall correctness score achieved, meaning that participants who took more time to read the documentation did not automatically achieve better results. As far as we know, this empirical study is the first analysis that explores the influence of security annotations in component diagrams to guide software developers when assessing microservice system security.

## Introduction

Microservice architectures are a widely applied software design concept to create highly scalable distributed systems by composing larger systems of lightweight, autonomously running services (Sill 2016; Dragoni et al. 2018). Designing robust microservice architectures is not trivial – with the growing number of services, their interaction also results in complex communication structures that are often not easy to track and follow (de Toledo et al. 2019). The situation is aggravated by the fact that microservices are often distributed in public cloud infrastructures to reduce hardware maintenance costs (Taibi et al. 2018). However, making microservices APIs publicly available poses a considerable security risk as now every potential attacker would gain direct access to the business logic and data provided through the services (Yu et al. 2019; Bermbach and Wittern 2020). Therefore, safeguarding and fortifying APIs to prevent unauthorized access is a crucial quality requirement.

Several guidelines and best practices exist to implement secure microservice communication (Pereira-Vale et al. 2019; Dias and Siriwardena 2020), along with a variety of microservice-specific recommendations from industry organizations such as NIST (2019); OWASP (2021), or the Cloud Security Alliance (2020).

To implement secure systems and ensure that they remain secure throughout the development and production cycles, developers must identify and understand the various security tactics used throughout their systems. However, understanding these security tactics can be challenging, especially for developers new to microservice architectures. This difficulty is exacerbated by limited or outdated system documentation (Zhou et al. 2017; Wen et al. 2019), often due to the fast-paced nature of development cycles (de Toledo et al. 2019).

Extracting pertinent information directly from the source code is a non-trivial task due to the intricate and diverse nature of these systems, which requires a wide familiarity with different programming languages and technologies (Newman 2021).

From an architectural perspective, the complexity of highly distributed systems primarily resides in their communication mechanisms and the interaction among services, which are often insufficiently represented in the source code (Silva et al. 2021). Additionally, when the emphasis is placed on developing customer-facing features that generate direct revenue, the availability of company-wide resources for developing and auditing secure microservice communication may be limited (Stocker and Zimmermann 2021).

To address these challenges, employing a higher level of abstraction to visualize the entire system can effectively eliminate extraneous implementation details and enable developers to focus on the security concepts employed and their associated implications without becoming entangled in the intricate web of various technology stacks. Therefore, providing architects with additional information and insights about the security features and tactics used within a microservice system could deliver tangible benefits to understanding these systems better and help avoid potential security risks.

Our current research focuses on creating such additional supportive visualizations – an architectural decomposition model we generate semi-automatically by parsing static artifacts, like source code, of microservice systems (Zdun et al. 2023b, a). The generated model visualizes the communication structures between services and provides additional security annotations to describe the security tactics implemented by each service and its API calls. For this, we identified several security tactics. We grouped them under four main security categories: *Secure Communication*, *Identity Management*, *Access Management*, and *Traffic Control*. We incorporated the tactics’ detection into our model generation process (Zdun et al. 2023b, a), together with a set of pre-calculated security metrics. This way, architects can quickly identify which communication paths are adequately secured or where the systems have security deficits without being restricted to possible outdated and informal system documentation or being forced to read through several hundreds of source files.

Although relying upon textual documentation can often result in ambiguous understanding and is open to interpretation, it tends to be the norm among developers due to its ease of creation and quick accessibility. Many developers prefer its use over standardized diagrams, like components or other UML diagrams (Ozkaya 2018). Some concerns regarding UML diagrams are the steep learning curve for reading and understanding the diagram semantics and the additional timely effort required to create these diagrams (Jongeling et al. 2022). Other voices argue that, while more formalized described diagrams may require more effort to understand and create them, they allow for better tool support and automatic processing (Hasselbring 2018).

We thus conducted an empirical study to check whether our annotated decomposition models affect the understanding of software architectures compared to informal documentation. As part of our experiment, we wanted to validate whether our models help the developers to understand better the security tactics used in a given architecture and, in case there is an improvement, if studying the enhanced documentation requires more time, as postulated by some researchers.

The results of our study provide a contribution to the discussion of whether the use of more structured and standardized diagrams is justified and provides enough benefits regarding their complexity compared to informal documentation, especially in such an essential field as security.

The study involved 70 participants, predominantly computer science students, and ten software experts with several years of industry experts, who were assigned to identify the security protocols used in two open-source reference microservice architectures and respond to comprehension queries to demonstrate their grasp of the applied security framework.

While the Experiment Group was provided with additional support through our security-annotated decomposition models and metrics, the Control Group relied solely on the textual and graphical documentation available for the systems. By comparing the performance and comprehension of the two groups, we aimed to evaluate the effectiveness of our annotated decomposition models in enhancing developers’ understanding of security tactics in a microservice system.

This experiment is designed to explore and discuss the following questions:

**RQ1**: *How do security-annotated component diagrams aid engineers in understanding microservice security architecture?*
**RQ2**: *How do security-annotated component diagrams affect the time required to understand security aspects of a microservice system?*
**RQ3**: *Do participants achieve better results when spending more time reading the documentation and answering the questions?*
**RQ4**: *How well did the participants perceive the component diagrams and metrics and is there a noticeable difference in the use of the materials?*
**RQ5**: *Do our diagrams and metrics benefit users differently based on their industry experience and compensate for limited expertise?*

The results show that providing participants with security-annotated component diagrams and metrics significantly improved the accuracy of their answers by up to 16%. Meanwhile, the participants did not need additional time to process the provided information. Both groups took nearly the same time to read the documentation and answer the questions. Throughout all groups, participants spent roughly one-third of the time consulting the documentation and two-thirds for the actual survey. Our findings further showed that novice developers benefit most from additional diagrams. Conversely, this benefit diminishes for seasoned developers, whose extensive knowledge allows them to assess systems effectively without supplementary materials. The results also reveal that while the participants widely appreciated the diagrams and confirmed that they improved their understanding of the security tactics, the additional architectural metrics were considered of limited benefit.

Although our process requires upfront implementation costs for reconstructing the architectural models, the benefits far outweigh its costs, especially considering that once the detectors for extracting the architecture are written, the updated models can be regenerated at any time with nearly zero costs.

To our knowledge, our study represents the first of its kind, examining how annotated component diagrams affect the understanding of security tactics in microservice architectures. Our findings can assist companies in supporting novice developers better and guide the development of more effective tools and strategies for assessing complex microservice architectures.

The remaining article is organized as follows: Section 2 examines previous and related work in this area. Section 3 contains background information regarding microservices and describes the security tactics we collected in our previous research. Section 4 details the experiment we conducted to evaluate the benefits of the component diagrams annotated with our tactics in detail, covering our research questions, the experiment execution, and the presentation and discussion of our results. Possible threats to validity are discussed in Section 5. Section 6 concludes the article. Additional descriptive statistics and demographics are added as an appendix in Section 7.

## Related Work

### Architectural Reconstruction Of Microservice Systems

Several strategies have been suggested for architecture reconstruction, including microservice-specific approaches such as those proposed by Rademacher et al. (2020), who use different viewpoints to reconstruct a microservice architecture, and Granchelli et al. (2017) who use docker files to automatically generate a model abstraction of a microservice system. A manual architectural recovery approach to define a microservice meta-model is presented by Alshuqayran et al. (2018), which they applied to eight different systems. Our previous work addresses the reconstruction of microservice systems in the face of polyglot programming, persistence, and technologies (Genfer and Zdun 2021; Ntentos et al. 2021).

### Architectural Reconstruction in the Context of System Security

Previous approaches for security reconstruction include using the Bauhaus tool to build security views (Sohr and Berger 2010), extracting security patterns (Bunke and Sohr 2011), or automatically extracting the architectural design of an application using a tool called *DelDroid* (Hammad et al. 2019).

When looking for approaches to enrich architectural models with additional security-related aspects, Lodderstedt et al. (2002) work on SecureUML can be considered a well-regarded contribution: They extended regular UML by defining a new role and authorization-based profile. By modeling architectures with this profile, the resulting system incorporates various security features like a role model and access control. However, a main difference between their technique and ours is that they create their models upfront and generate the code afterward. In contrast, we decompose our models from a system’s code base. Another approach that uses security annotated graph structures is the *Scoria* tool, proposed by Vanciu and Abi-Antoun (2013).

With their tool, the authors intend to support security architects when analyzing security risks in runtime systems. They achieve this by creating an object graph and further annotating it with additional security properties. While their general approach is similar to our research, they rely on a more semi-automatic process where software architects manually add annotations to the graph. In contrast, we automatically extract our security tactics with our detectors from the underlying code.

El-Attar et al. (2015) even go one step further by not enriching a model with annotations but defining a new set of notations for expressing security concerns within UML state charts. They also conducted an empirical study to evaluate whether their new notations improve the understandability of security features within a given system. The results of their study allow the conclusion that while their new notation decreased the time the participants took to read the models, there needed to be statistical significance regarding the accuracy when interpreting the models. These results differ from ours: Our annotated component diagrams significantly improved the participants’ answers’ correctness while leaving the reading or response time untouched. However, these results are barely comparable due to the different contexts and experiment setups.

### Empirical Studies on Software Systems

There is also a variety of empirical studies regarding software systems and security. Allodi et al. (2020) conducted a controlled experiment with students and professionals to evaluate the effects of practical experience and formal education on assessing system vulnerabilities. Another study by Votipka et al. (2020) analyzed the vulnerabilities of 94 submissions to a secure coding contest. Their results indicate that most security exposures happen through developers misunderstanding the security concepts they use. Labunets et al. (2014) investigate the effect of visual vs. textual methods to assess security risks. While both methods achieved similar results, participants perceived the visual approach as more helpful. Our observations come to a similar conclusion. Nasab et al. (2023) collected 28 security practices and validated their usefulness through a survey with 74 participants they recruited online. The approach we chose for our controlled experiment is similar to those used in other studies: Paulweber et al. (2021) use a controlled experiment to examine the understandability of different language contracts like *mixins*, *interfaces*, and *traits*. Parts of our setup are based on their procedure, as we also track the same dependent variables like Correctness and Response Time to measure the performance of our participants. However, in contrast to their approach, we use a within-subject design where each participant is part of the control and experiment group. Czepa and Zdun (2018) and Czepa et al. (2017) investigates the comprehensibility of formal methods for specifying temporal properties. Compared to their experiments, our approach uses a *within-subject design* (Charness et al. 2012), thus eliminating the problem of having groups with an unbalanced distribution of knowledge or skills.

## Background

This section introduces some of the main concepts of microservice architectures and discusses the structure of the security annotated component models we provided as additional help during the survey.

### Microservices

Microservice architectures consist of small, autonomous service units, each encapsulating a specific business logic and acting independently from each other. They present the fundamental building blocks, which, when put together to form larger systems, allow the development of highly scalable and robust distributed applications (Newman 2021). Some essential tenets distinguish them from previous practices in building distributed systems, like being implemented with polyglot technology stacks and programming languages, using cloud-native technologies, lightweight containers, and continuous delivery pipelines (Thönes 2015).

Communication between individual services happens via lightweight protocols like synchronous HTTP/HTTPS or message-based techniques for asynchronous communication. The main access point for every communication attempt is their public API (Dragoni et al. 2017), commonly implemented using a protocol supporting a REST-based approach, like HTTP or its more secure alternative, HTTPS. Encrypted transport protocols should always be preferred if an API call might contain sensitive information (Dragoni et al. 2017).

A common practice in microservice development is to group these public APIs behind a single entry point, the *API Gatewa*y. This gateway works as a reverse proxy and controls access to the underlying microservice APIs. This approach can help improve the system’s security, as the gateway controls all incoming calls and can also implement various security measures (Taibi et al. 2018).

### Microservices Documentation

Documenting microservices is challenging due to the rapid evolution of these systems and their polyglot nature, often resulting in manual documentation efforts that are difficult to keep in sync with the actual state of development (de Toledo et al. 2019). This discrepancy may sometimes lead to incomplete or ambiguous documentation (Kleehaus and Matthes 2019). While different approaches have been suggested for documenting APIs, the OpenAPI specification^1^ established itself as the current defacto standard, allowing developers to describe and document their APIs in a language-independent way (Da Rocha Araujo et al. 2022). This approach further allows automatic processing, e.g., to generate client API interface stubs. When using OpenAPI in such a documentation-first workflow, it is also ensured that the code always stays in sync with the current API version. However, even the use of OpenAPI has limitations: Currently, only REST APIs are supported by the specification, with the asynchronous counterpart AsyncAPI^2^ not being widely accepted yet. Also, generating the API stubs from the documentation requires additional tool support, which may not be available for all languages and features. Falling back to manually implementing the code based on the specification again bears the risk of documentation and code getting out of sync.

To provide our Control Groups with realistic conditions regarding API documentation, we provided them with printed excerpts of the online documentation of our reference systems, which, while still having informal character, should be sufficiently up-to-date to solve our comprehension tasks.

### Component Diagrams

Despite relying solely on source code artifacts, abstract component diagrams provide a more accessible approach for analyzing the communication structure within a microservice system. The information density of code is relatively high and requires knowledge of all the programming languages and technologies used. Instead, component diagrams approach this problem more abstractly and thus focus on the essentials without getting lost in the details.

**Fig. 1:**
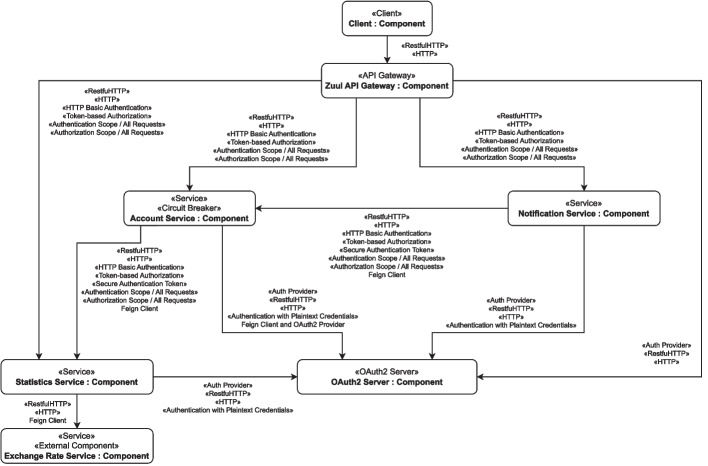
Component diagram with security annotations generated from the *Piggy Metrics* open-source application

To describe a microservice system more formally by a component diagram, we rely on the definitions from our previous research (Zdun et al. 2023b), where we express a microservice architecture as a directed graph consisting of nodes representing components and edges defining the connections between them. These components represent specific architectural entities such as *Services* or *Clients* and can even have more specific roles within the architecture, like modeling an *API Gateway* or enabling *Service Discovery*. For this, additional stereotype annotations express the component’s function. Connectors between components model interactions, such as asynchronous communication, authorization means applied during an interaction, or HTTP-based service calls. A directed connection from *Service A* to *Service B*, for instance, expresses a communication flow (an API call) from *Service A* to *Service B*. Similar to components, we also use stereotypes to describe a connector’s roles within the system or specify other essential aspects of the communication (e.g., the protocol used for the data transfer). Based on this connotation, we further define our decomposition model as a tuple consisting of the total number of components and their connections within the system. Together, these components and connectors can represent all available types and roles these components and connectors can represent (Zdun et al. 2023b). See Fig. 1 for an example of such a component model.

Reconstructing such component models was part of our previous work (Zdun et al. 2023b), where we used a lightweight parser approach (Ntentos et al. 2021) to identify architectural elements out of underlying source code and use them to rebuild a component-based structure of our system. Our approach assumes that experts familiar with a given system can identify the main concepts and technologies used for the implementation. In contrast to other existing reconstruction methods that aim at creating a complete architectural reconstruction of a system, we use this expert knowledge to develop small, language-agnostic parsers, called detectors, that isolate only concepts in the source code that we consider essential for our research and abstract these into the components and connectors mentioned above.

After these detectors are implemented, the reconstruction process can run automatically without any manual interaction, making it exceedingly feasible for automated processes like continuous integration pipelines or tracking such systems’ evolution (Zdun et al. 2023b).

While some of our detectors may be tailored to specific concepts, their reuse among different projects is possible as long as the^3^ same concepts are used.

Besides reconstructing the basic structure of each microservice, we also developed detectors to identify various security features within the architecture (Zdun et al. 2023a) and use this information to add additional annotations to our previously generated component diagram.

The security annotations used throughout the diagram will be explained further in the next section.

### Security Tactics

Defining a microservices architecture that supports secure communications can be challenging. While there exist guidelines on how to implement secure systems, conforming to all these best practices is not easy, as many other forces drive architectural changes, e.g., providing customers with new business functionality (Stocker and Zimmermann 2021).

Applying security tactics is a way to ensure that an architecture conforms to security expectations.

By reviewing existing security guidelines, the gray literature, including open source projects, online discussions or blog posts, and the scientific literature (Pereira-Vale et al. 2019; Dias and Siriwardena 2020; NIST 2019; OWASP 2021; Cloud Security Alliance 2020), we identified a catalog of security tactics as options to drive architectural design decisions (Zdun et al. 2023b, a). To further refine and validate these tactics, we asked selected industry experts to review and assess our tactics based on the standards they applied as part of their work in various industrial projects.

After defining our set of security tactics, we grouped them into four widely used categories:

#### (1) Secure communication

This tactic combines all measures used to secure the data transfer between services. Regarding the secure communication (of the API), the following options are available:

- **Encrypted Communication**: Encrypted protocols such as SSL/TLS or HTTPS are used for communication on a connector.
- **Unencrypted Communication**: Unencrypted protocols such as HTTP are used for communication on a connector.

If sensitive data is transferred between APIs, the literature strongly advises against the use of *Unencrypted Communication* (NIST 2019; Cloud Security Alliance 2020).

#### (2) Identity management

This category summarizes all tactics used to validate and verify the identity of users. We identified seven different authentication strategies:

- **Token-based Authentication**: Authentication is performed using cryptographic identity tokens such as SAML^4^, OpenID^5^, or JWT^6^ tokens.
- **Protocol-based Authentication**: Authentication is performed based on the features of an encrypted communication protocol, such as SSL- or SASL-based authentication.
- **API Keys**: Authentication is performed based on API Keys, i.e. authentication with a unique token per client that the client can present to the API endpoint for identification purposes (Zimmermann et al. 2023).
- **Plaintext-based Authentication**: Authentication information is transferred as plaintexts, such as in HTTP Basic Authentication, form-based authentication, or homegrown implementations.
- **Plaintext-based Authentication over an Encrypted Protocol**: Authentication information is transferred as plaintext over a secure (i.e. encrypted) communication protocol such as TLS/SSL.
- **No Authentication**: No authentication method is used.
- **Authentication Not Required**: The connector does not need any authentication. This is, for instance, the case if a public API is offered that any client can access without restrictions.

*Token-based Authentication* and *Protocol-based Authentication* are recommended practices. Regarding *Identity Management*, we consider secure authentication crucial when accessing any part of a microservice system. This is especially true for all APIs directly accessible through client applications. However, even non-secure authentication tactics may be beneficial compared to using *No Authentication*.

#### (3) Access management

Like *Identity Management*, *Access Management* controls the authorization process and checks user or service access rights after verifying their identity. Accordingly, *Access Managment* related security tactics are similar but focus on authorization aspects instead.

- **Token-based Authorization**: Authorization is performed using a cryptographic access token issued by a central access management server, such as an OAuth 2.0 token.
- **Encrypted Authorization Information**: Some other kind of encrypted authorization scheme is used, but not with a standardized central access management server.
- **Plaintext Authorization Information**: Authorization information is transferred as plaintext.
- **Plaintext-based Authorization Information over an Encrypted Protocol**: Authorization information is transferred as plaintext over a secure (i.e., encrypted) communication protocol such as TLS/SSL.
- **No Authorization**: No authorization method is used.
- **Authorization Not Required**: The connector does not need any authorization. This is, for instance, the case if access is allowed for each identified client or in public APIs with no access restrictions.

While *Token-based Authorization* and *Encrypted Authorization Information* are recommended practices regarding *Access Management*, any type of authorization should be preferred compared to *No Authorization* (Pereira-Vale et al. 2019).

#### (4) Traffic control

Controlling the traffic to and from a microservice system can be achieved by placing additional network components in front of the service APIs that work as facades and shield the system from direct client access. Such facades act as reverse proxies by routing client requests to backend services. They can also realize cross-cutting concerns such as authentication, authorization, SSL termination, or several monitoring tasks. The most widely used pattern for this is the API Gateway (Taibi et al. 2018), but variations and homegrown solutions exist, too.

- **API Gateway** provides a single endpoint for the clients and internally maps the requests to backend microservices.
- **Backends for Frontends** is a variation of API Gateway that defines a separate gateway for each kind of client, e.g., a Web app, a mobile app, and a public API gateway.
- **Frontend Service**: While the prior options usually use dedicated technologies for establishing traffic control, some systems use a homegrown frontend service that acts as a Facade for other services in the system. It can only offer the traffic control features built into that service.
- **Direct Access from Clients to Services**: Clients access system services directly; thus, no traffic control is provided.

From a security perspective, if all potential API traffic is managed by *API Gateway*, *Backends for Frontends*, or *Frontend Service*, this security feature is realized. *Direct Access from Clients to Services*, in comparison, is only a sub-par solution when realizing *Traffic Control*.

We incorporate these security tactics into our component models using additional annotations to give developers hints on which security features are implemented or missing. In that way, we can mark connectors with their corresponding security tactics, such as encrypted communication, or specify which type of authentication is required to access an API endpoint. Figure 1 illustrates one of the component models we used in the experiment surveys, annotated with all identified and essential security aspects.

### Architectural Metrics

We also use our models to derive a set of architectural metrics by giving architects another tool to assess significantly larger microservice systems, where visual representations can quickly become confusing (Stevanetic et al. 2014). Our metrics are calculated based on the results provided by the detectors we use to identify various security features (see Section 3.4). If we take, for instance, the metric for determining the ratio of the *Secured Distributed Connectors* (*SDC*), we see that this metric divides the results delivered by the detector responsible for identifying all encrypted connectors (i.e., connections that use the HTTPS protocol) by the total amount of all distributed connections (all connections that are not of the type *InMemoryConnector*):

#### Secure Distributed Connector (SCO)

$$ SCO(cn) =\frac{secure\_connectors(distributed\_connectors(cn)).successful}{distributed\_connectors(cn))} $$

where *cn* represents the set of all available connectors within the systems (Zdun et al. 2023b). The value calculated as a result of this metric lies between *0* and *1*, where *0* indicates the absence of any secured connector, and a value of *1* expresses the ideal case that all connector use encryption. This behavior generally applies to all our metrics, with their values ranging from *0* (lack of any support for the feature) and *1*, indicating full support.

To validate our metrics, we selected ten open-source microservice systems and reconstructed an architectural model out of each system. Additionally, we created models from 20 additional variations of these systems, where specific characteristics like the type of communication or the usage of components like an API gateway were altered. In the next step, we calculated our metrics for every system and, at the same time, let industry experts assess manually whether and to what degree these systems apply our security tactics. Using ordinal regression analysis, we evaluated how well our calculated metrics correlate with the experts’ judgment (Zdun et al. 2023b).

The main goal of our metrics is to help drive the decision process as they provide an easy and fast way to identify specific deficits in the secure communication of a system. Table 1 illustrates a subset of our metrics catalog with already precalculated example values for the component diagram we generated out of the *Piggy Metrics* reference application (see Fig. 1). In general, value ranges for each metric are between *0* and *1*, with *1* indicating full support and *0* indicating the lack of any support.

## Experiment

### Research Questions and Hypotheses

To evaluate the usefulness of our auto-generated annotated software decomposition models when assessing microservice architectures, we conducted an experiment where we compared our diagrams against informal textual and visual system documentation.

For this, we measured the level of our participants’ understandability during the experiment by two dependent variables:

- The Correctness achieved by the participants when answering the questions, either by marking the correct multiple-choice answers or by giving the correct numerical or textual answers. Values could be between 0 for a completely incorrect result and 1 for an absolutely correct answer, with higher values obviously indicating a better comprehension of the system. A detailed description of how the Correctness was calculated is given in Section “Data-set Preparation”.
- The Response Time it took the participants to complete the questionnaire, expressed in minutes. We instructed the participants to measure the time they needed to answer each question and also asked them to record the time they spent reading the documentation. The higher the Response Time, the more time the individual took to read the documentation and answer the question.

**Table 1:** Selected Architectural Metrics related to Security Tactics (Zdun et al. 2023b, a) and their calculated values for the *Piggy Metrics* microservice system.

Metric	Description	Value
*Secure Distributed Connector Ratio (SCO)*	The SCO metric sets the secure distributed connectors in relation to all distributed connectors.	0.03
*Secure UI Connectors Ratio (SUC)*	Sets the secure connectors originating in the UI in relation to all connectors in this set.	- (no UIs)
*Securely Authenticated Connectors on Client/UI to Service Path Ratio (AEC S)*	Securely authenticated client/service path connectors requiring authentication in relation to the total number of client/service paths connectors requiring authentication.	0.4
*Authorized Connectors on Client/UI to Service Path Ratio (AUC)*	Ratio between authorized connections from client to services against the number of all client-service connections. Measures the overall authorization level between clients and services.	1.0
*Gateway Paths Ratio (GWP)*	The GWP metric calculates the ratio of client/service paths containing a gateway to all such paths.	1.0
*The Frontend Service Paths Ratio (FEP)*	The FEP metric calculates the ratio of client/service paths containing a frontend service to all such paths.	0.0

We hypothesized that using our security annotated component diagrams as supplementary material gives the participants a significant advantage, both regarding their Correctness and Response Time when assessing the security tactics used in a microservice architecture, compared to using only informal textual and visual documentation.

Based on this assumption, we derive our first two research questions:

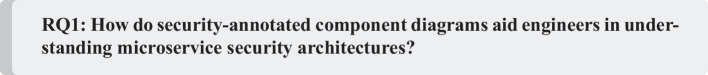



We postulated a null hypothesis and an alternative hypothesis focused on the participant’s Correctness values to answer this question.

${{\textbf {H}}_{0,1}}$: The Correctness in answering the questions shows no difference when using security-annotated component diagrams as additional help.
${{\textbf {H}}_{A,1}}$: Using additional security-annotated component diagrams when answering the questions has a significant effect on the Correctness the participants give, compared to relying solely on informal documentation.

We also wanted to find out whether the component diagrams may affect the overall time participants need for their assessment, leading to our second research question:

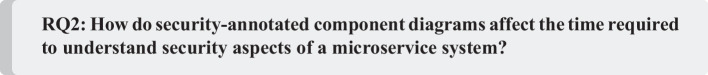



Again, we propose a null hypothesis and an alternative hypothesis focusing on the Response Time variable:

${{\textbf {H}}_{0,2}}$: The Response Time the participants needed to answer all questions did not change significantly when using additional component diagrams.
${{\textbf {H}}_{A,2}}$: The Response Times of the participants were significantly different when using our component diagrams to answer the survey questions.

While both variables provide meaningful insight into the comprehension the participants have about the system, looking at each variable in isolation can also give a slightly distorted picture: A candidate could, for instance, give only wrong answers to improve their response time. Likewise, taking a disproportionately long time to read the documentation could improve Correctness but be impracticable in most real-world projects due to time and budget constraints. To consider this, we use a combination of both variables to measure the participants’ level of **Understandability** on the subject, as it has already been done by other studies (Feigenspan et al. 2013; Hoisl et al. 2014), including our previous work (Czepa and Zdun 2019; Paulweber et al. 2021).

When evaluating the effect our approach has on understandability, one reasonable conjecture would be that the more time participants spend reading the documentation and answering the questions, the better results they achieve, we therefore define our third research question as follows:

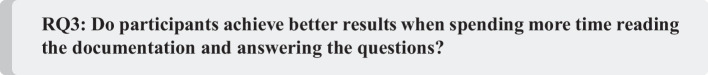



To answer this question, we first postulated the following null hypothesis and corresponding alternative hypothesis for the group **without access to our additional component diagrams**:

${{\textbf {H}}_{0,3}}$: The Correctness of the answers does not improve significantly with increasing or decreasing Response Time for participants using only the provided informal textual and graphical documentation without additional component diagrams.
${{\textbf {H}}_{A,3}}$: There is a significant effect on the Correctness when the Response Time increases or decreases for the group that uses only the regular documentation without any additional material.

Secondly, we also postulated corresponding hypotheses for the group **with access to our security annotated decomposition diagrams**:

${{\textbf {H}}_{0,4}}$: Longer Response Times do not result in significantly higher Correctness values, even if the participants have access to additional software decomposition models annotated with security tactics.
${{\textbf {H}}_{A,4}}$: A significant effect on the Correctness values is visible when the Response Time increases or decreases for the group that uses additional decomposition models.

To explore which materials, diagrams, or metric tables helped the participants the most, we used a self-assessment questionnaire to gather feedback on their performance and the support provided by the materials. This led to our fourth research question:

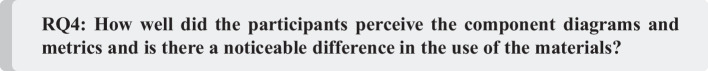



Furthermore, we investigated the effect of a subject’s working and industry experience on the results and whether using our additional diagrams may compensate for missing experience. Answering this question can give hints on how novice developers could be better supported in understanding the security mechanisms of distributed systems.

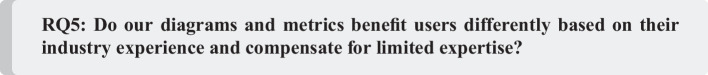



To our knowledge, our study is the first to evaluate the effects of security-annotated component diagrams on understanding security features, and our results provide interesting insights for the research community and the industry.

### Experiment Design

#### Setup

The structure of our study is based mainly on the guidelines proposed by Jedlitschka et al. (2008) and Kitchenham et al. (2002) regarding study design in empirical software engineering. To make our statistical evaluation and data visualization more robust, we also followed the principles suggested by Kitchenham et al. (2017), e.g., by using a combination of different statistical diagram options and using robust statistical methods. We conducted our study as a controlled experiment with 60 participants from universities with backgrounds in computer science studies, either enrolled or graduated, and 10 subjects we recruited from industry, leading to a total of 70 participants.

As part of the experiment, our participants had to evaluate two open-source reference microservice architectures, the *eShopOnContainers*^7^ project (*ES*) and the *Piggy Metrics*^8^ architecture (*PM*). We created two equivalent versions of our survey: Subjects at the universities received it in written form as a hands-on task, while the industry experts were invited to conduct the same survey as an online questionnaire.

Both groups received printed excerpts of each system’s online documentation (either in written form or as a PDF) and a survey containing several questions focusing on certain security aspects regarding the system’s architecture. There were three types of questions participants had to answer:

1. textbfMultiple choice Participants were presented with up to five statements about the system’s security features and had to choose all the correct statements. There was always at least one correct option, but multiple correct ones were also possible. The following is an example from the questionnaire with the correct answer already selected.
  $\Box$: The support for Secure Communication on links between API Gateways/BFFs and API Services is acceptable.
  $\Box$: There is some support for Secure Communication on links between API Gateways/BFFs and API Services, but also there are serious flaws with regard to Secure Communication in this context.
  $\Box$: The Identity Service is the only API service using acceptable Secure Communication on its links to API Gateways/BFFs.
  ☑: The support for Traffic Control is acceptable as all API traffic goes through an API Gateway/BFF.
  $\Box$: The support for Traffic Control has serious security flaws as API Gateways/BFFs can be bypassed.
2. **Cloze** These questions were primarily related to the system’s structure. Students had to fill a gap in a text with a correct numerical value they had to derive by consulting the system’s documentation:
  There are 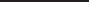 number of links that connect Clients or User Interfaces (UIs) to API Gateways/BFFs via RESTful HTTP.
  There are 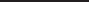 encrypted communication and authenticated links between API Gateways/BFFs and API Services.
  The Identity Service is accessed by 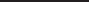 other components.
  In this case, the correct answers would be *3*, *0* and *4*.
3. **Free Text** To answer the last type of question, the students had to demonstrate their knowledge of the system’s interconnection and relations between its components. For this, participants had to enumerate the communication paths that meet a given condition by naming all components of these paths. *List the links between API Gateways/BFFs and API Services that are authorized using a secure method.* Answering this question correctly would require the participants to name the following connections:
  Mobile Gateway $\rightarrow$ Basket Service
  Mobile Gateway $\rightarrow$ Ordering Service
  Web Client Gateway $\rightarrow$ Basket Service
  Web Client Gateway $\rightarrow$ Ordering Service
  The component names did not have to match exactly, as, for instance, answers like *Basket Service*, *Basket Domain Service*, or even just *Basket* were all considered correct, and were transcribed into a standardized during evaluation.

The questions were constructed so that answering them should be possible by consulting the provided documentation. However, extracting all relevant information from the textual description could be tricky, as sometimes the information might have been imprecise or ambiguous – a scenario not uncommon when relying on informal or manual documentation (Kleehaus and Matthes 2019).

**Fig. 2:**
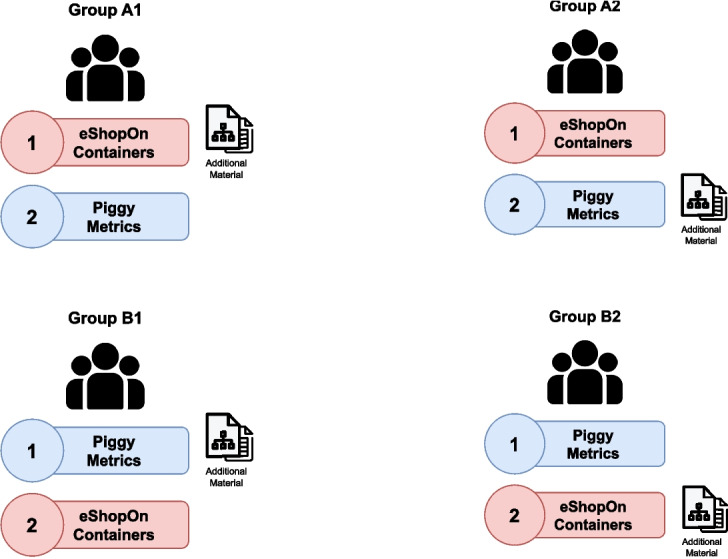
The participants were organized in four groups with alternating setups to avoid learning effects or unequal distribution of participants’ skills

While the Control Group had to rely solely on the provided documentation to answer all questions, the Experiment Group received additional component diagram drawings and a table summarizing a selection of precalculated architectural metrics. The components and connectors in the diagrams contained annotations describing the most relevant security features used throughout the projects. Every student had to analyze both example projects but received additional help only for one of the two examples. This way, each participant served as a member of the Experiment Group for one task while at the same time being part of the Control Group for the other task (*Within-Subject design* Charness et al. 2012). This approach minimizes potential bias created by an unequal distribution of certain factors – such as experience or years in the industry – between the two groups. We also varied the order of the example architectures to avoid bias through learning effects, resulting in four different group setups, as depicted in Fig. 2.

#### Variables

##### Dependent and independent variables

Our experiment consists of two dependent variables, Correctness and Response Time, which we use to study the Research Questions **RQ1** and **RQ2** - and, in combination, also for **RQ3**. As mentioned in Section 4.1, Correctness measures the participants’ overall result when answering the questions. Response Time counts the overall time they took to complete the survey, including the time for reading the documentation and answering the questions.

Besides that, we have a single independent variable: the participant’s membership to either the Experiment or Control group, depending on whether they have received the additional material during the survey. Determining the effect of this membership on the participant’s Correctness and Response Time is the main contribution of our experiment.

##### Control of confounding variables

We applied different measures to rule out several confounding factors affecting our experiment’s result: To ensure that our participants did not use any external information sources, we conducted the study as a closed-book exam where no additional resources except our diagrams and metrics tables were allowed. Unfortunately, we could not ensure this for our industry experts since they processed the survey online without direct control. However, all our experts ensured they did not use any material supplementary to the one provided. We also ensured that each round of our experiment followed the same protocol to minimize variations, with a few adjustments to the experts’ survey.

Other critical confounding factors are a subject’s characteristics, like their programming experience or age. If these are not equally distributed among both groups, they can strongly distort the relation between the dependent and independent variables. To avoid this subject-level confounding, we use a within-subject experimental design where every person is part of the control group for one project and the experiment group for the other. In that way, we ensure that all individual characteristics are part of every group. This approach, however, can still introduce other confounding factors, such as the order in which the projects are handled or the varying complexity between them (Charness et al. 2012). To deal with the former, we introduced a randomized order assignment consisting of four groups, and for the latter, we carefully selected two projects with very similar structure and complexity based on our experience and some key indicators like the number of services.

#### Participants

**Fig. 3:**
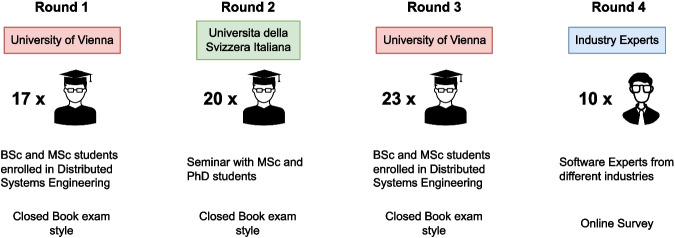
The experiment was conducted in four rounds at different universities and with selected industry experts

We repeated our experiment in four different rounds, which involved a total of 70 participants (see Fig. 3 for an outline of the procedure). The first three rounds were conducted with students from the University of Vienna and the Universitá della Svizzera Italiana (USI)^9^, Lugano. As for the last round, we invited several industry software experts from more than six countries, from whom we could verify their experience through personal contact or by analyzing their profiles on social network sites like LinkedIn. Due to easier organization, we switched to an online procedure, where participants could access the study materials using an invitation link and send their responses back after completing the questionnaire.

Although most of our participants were not professional software architects, the demographic data we raised verified that the students who participated in our experiment were a good substitute for professional software developers, as they already had a wide range of knowledge and experience. Compared to a 2016 study among 50,000 developers of the online platform *StackOverflow*^10^, where most developers had between two and five years of experience, our participants show a similar, but slightly more experienced demographic distribution (see Section “Demographics” for details). We therefore regard our students as a well-suited target audience for the experiment. Other studies on empirical software engineering come to similar results with respect to the use of students as substitutes for professional developers (see, for instance, Svahnberg et al. 2008 or Falessi et al. 2018). In addition, experimenting in two countries further diversified the participants’ profiles. The additional software experts who participated helped make our audience and thus our findings more generalizable.

#### Material

The main components of our study can be divided into two artifacts:

- **Information Sheet**: Participants received a document containing information about microservice systems and security tactics (similar to the background information in Section 3). This document was sent two weeks before the experiment started to ensure that all participants had at least the minimum background knowledge necessary to participate.
- **Experiment Survey**: The experiment survey consisted of four parts:
  1. The first part was an optional demographic survey asking the participants about their knowledge and experience in the fields relevant to the experiment.
  2. The survey consisted of two task descriptions, one per example project. Each project was described through a copy of its online documentation. We focused on the parts of the documentation that were most relevant for security aspects but added some less security-related information to make finding the relevant information more difficult. Depending on whether the participant was in the Experiment Group or not, the documentation was accompanied by an additional component diagram annotated with security-relevant features and a list of architectural metrics and their precalculated values (see Table 1 for an excerpt).
  3. Following the documentation was a list of seven tasks to assess the participant’s understanding of the security tactics implemented in the corresponding example. The students had to consult the provided textual and visual descriptions to answer all questions correctly.
  4. Each experiment and the overall survey concluded with a questionnaire where students had to self-assess their performance on a *Likert-Scale* (Joshi et al. 2015).

#### Example Projects

When looking for adequate and representative example architecture for an experiment, several factors, like the system’s complexity, the used technology stack, and the provided documentation, must be considered, as they can strongly influence the participants’ performance. While using real-world microservice architectures would provide the best insights, these systems often consist of several dozens or even hundreds of services, and using such architectures for a controlled experiment can be problematic as they require specific domain knowledge and more preparation time to become familiar with them. Also, analyzing such large systems within one and a half hours could be very demanding, especially for novice developers.

Therefore, we chose two mid-level open-source microservice reference applications, the *eShopOnContainers* and the *PiggyMetrics* project. While both projects are relatively small compared to actual industry systems, they apply many common patterns and best practices in microservice development, like an event-driven architecture and a good separation of concerns (Nordli et al. 2020). Both systems are also well documented, which was an essential criterion for our experiment, as we had to provide enough informal documentation for the study.

Also, the two systems are well received and frequently used by the academic community (Nguyen et al. 2019; Smith et al. 2023; Vural and Koyuncu 2021; Hu et al. 2020; Kirschner et al. 2023; Matos et al. 2023; Nasab et al. 2021; Morais et al. 2021).

##### eShopOnContainers

*eShopOnContainers*^11^ is Microsoft’s reference application showing how to implement a microservice architecture in .NET. The system comprises four domain or business services and several infrastructure services for user authentication or logging. While most backend services use an event bus for asynchronous communication, an additional API gateway layer handles the communication between the front and backend. Although the system’s size is manageable, its asynchronous communication gives it considerable additional complexity, especially regarding security-relevant aspects.

##### Piggy metrics

Our second reference system, *Piggy Metrics*^12^ is a mainly Spring-Boot-based microservice financial advisory application. It has three domain services communicating via a REST interface and uses several Java-based infrastructure technologies, like, for instance, Zuul^13^ as API gateway and Eureka^14^ for service discovery.

While both systems are comparable in their number of domain and infrastructure services, the *Piggy Metrics* architecture may be easier to access due to the more synchronous communication structure. However, we consider these differences to be almost negligible.

### Execution

The preparation and execution of our experiment followed the guidelines from the literature (Jedlitschka et al. 2008; Kitchenham et al. 2002).

#### Pretest

After defining our questionnaire and the corresponding information sheet, we organized pretests with tutors (students who work as teaching assistants). Like the actual participants later on, the tutors received the information sheet two weeks in advance to gain essential knowledge to solve the tasks.

During the pretest, each tutor was assigned to a random group. We had them answer the survey under the conditions intended later, i.e., 90 minutes to answer all questions and no additional help despite the provided documentation and component diagrams. We concluded with an open discussion and asked the tutors to describe their experience during the test, especially regarding the difficulty and available time.

One feedback we received during the pretest was that the time allotted for the experiment was tight: While the tutors could still complete the survey within the given timeframe, they said it was challenging. Therefore, we decided to reduce the total number of questions in the final survey.

#### Study Procedure

We announced the experiment at the University of Vienna during the regular DSE lectures. We distributed the preparation material (the experiment information document) through the university’s e-learning platform two weeks before the start date. These information sheets were also the only supplementary material permitted during the study. Similarly, at the Universitá della Svizzera Italiana (USI), the experiment was advertised as a seminar^15^ for Master’s students enrolled in a Software Architecture lecture but also open to participants from the entire faculty of informatics. The experiment was conducted at the universities in written and closed book exam style with only the information sheets as supplemental material. Questionnaires were handed out so that every four consecutive participants received different groups.

The industry experts participating in the online study were informed upfront via email or private message about the topic and the study’s modus operandi. They received the information sheet together with a link to the online form. The survey was implemented as a *Google Forms Sheet* 16 replicating the same content as the written version.

All answers were entered into a spreadsheet and exported as a comma-separated-value file for later processing. Multiple-choice and numerical answers were entered directly, while textual answers, i.e., names of services or communication routes, were transcribed into a standardized format to make them readable by our automated scripts.

### Results

In this section, we aim to answer the research question from Section 4.1 based on our statistical analysis of the gathered survey results. For a deeper demographical and descriptive analysis of the data set, please consult Section “Demographics” and Section “Descriptive Statistics” in the Appendix.

#### RQ1 & RQ2: Testing for Significance

Research questions **RQ1** and **RQ2** postulated four hypotheses about whether participants with our additional supplemental material achieved different results than those in the control group. To verify these hypotheses and thus answer the corresponding research questions, we executed significance tests for our dependent variables, Correctness, and Response Time.

Since we assume our dataset is non-normally distributed (see Appendix “Testing for Normal Distribution”), we focus our selection on non-parametric tests: Kruskal and Wallis (1952) and Wilcoxon (1992) are two established and commonly used non-parametric rank-sum tests, but both appear to be less robust against heterogeneous variances (Kitchenham et al. 2017; Zimmerman 2000), and should, therefore, be used with caution. Instead, more robust approaches like Cliff’s $\delta$ (Cliff 1993) or the Brunner Manzel test (Brunner and Munzel 2000) should be used instead instead (Kitchenham et al. 2017; Karch 2021; Neuhäuser et al. 2007).

Cliff’s $\delta$ is an effect size measure that cannot only estimate whether a difference between two groups is significant, but it can even express how big this difference is. It does this by estimating the probability that a selected value from one group is larger than a value from the other, expressing the measure of dominance between the two groups (Macbeth et al. 2011; Cliff 1993). While this approach makes it particularly well suited for discrete ordinal data, it also works for continuous data as our Correctness and Response Time (Delaney and Vargha 2002). To calculate the relevant Cliff $\delta$ values, we used the *Ordinal Dominance Statistics* R-package^17^.

The results for the *eShopOnContainers* project are summarized in Table 2 for Correctness and Table 3 for Response Time. For completeness, we also report the Kruskal-Wallis and the Wilcoxon Rank Sum test values, but our analysis focuses on the values gathered through Cliff’s $\delta$.

**Table 2:** Significance Test Results for **Correctness** – *eShopOnContainers*

Correctness (ES) - Control vs Exp.		
Cliff’s $\delta$	$\delta$	0.48
	$P(X < Y)$	0.72
	*p*	0.001
Kruskal-Wallis	*p*	0.001
Wilcoxon Rank Sum	*p*	0.001

**Table 3:** Significance Test Results for **Response Time** – *eShopOnContainers*

Response Time (ES) - Control vs Exp.		
Cliff’s $\delta$	$\delta$	0.12
	$P(X < Y)$	0.544
	*p*	0.545
Kruskal-Wallis	*p*	0.422
Wilcoxon Rank Sum	*p*	0.426

The eponymous delta value of Cliff’s $\delta$ measures to what degree the two groups overlap; values close to 1 or -1 indicate that both groups are not overlapping, while values closer to 0 represent completely overlapping groups (Macbeth et al. 2011). Accordingly, the closer the effect size is to -1 or 1, the more significant the difference between the two groups is. Hence, the value of 0.48 we measured for our Correctness in the *eShopOnContainers* project indicates that our groups do partly overlap. The next value, $P(X < Y)$, expresses the probability that a random sample taken from the Control Group has a lower value than a sample from the Experiment Group. Here, our probability of 0.72 again supports our previous observation that the Experiment Group performed slightly better than the Control Group. The *p* value in Cliff’s $\delta$ section gives us a final hint of whether the groups’ differences are significant: Since our *p* value (0.001) is lower than the assumed $\alpha$ (0.05), the difference between the Control and the Experiment is significant. This means that for the *eShopOnContainers* project, we can reject our null hypothesis ${\textbf {H}}_{0,1}$ and accept our alternative ${\textbf {H}}_{A,1}$ instead.

In contrast, the results for Response Time we measured for the same project look somewhat different: Here, we have a $\delta$ value closer to 0, indicating that both groups strongly overlap, making significant differences unlikely. Accordingly, the chance of picking a higher value from the Experiment Group is almost 50:50, which indicates that both groups show remarkably similar characteristics. And, finally, we have a relatively high *p* value that further supports our assumption that there is no significant difference between the two groups regarding the recorded Response Times. Based on these results, we cannot reject our null hypothesis ${\textbf {H}}_{0,2}$ for this project.

Looking at the results for *Piggy Metrics* (Tables 4-5), the situation presents itself similary: While the $\delta$ value of 0.272 for Correctness is slightly lower than we observed in the previous project, it still indicates that both groups may differ significantly. Also, the probability of catching a higher value from the Experiment Group (expressed by $P(X<Y)$) is above 50%. With a *p* value of 0.048, which is still below our $\alpha$ threshold, the two groups are distinguishable. However, the difference is less substantial this time than for the previous two groups. It may be interesting to note here that the industry experts improved the results of the Control Group, leading to a convergence of both groups. Still, the combination of all three parameters allows the conclusion that we can also reject our null hypothesis ${\textbf {H}}_{0,1}$ in favor of the alternative ${\textbf {H}}_{A,1}$ for *PiggyMetrics*.

**Table 4:** Significance Test Results for **Correctness** – *Piggy Metrics*

Correctness (PM) - Control vs Exp.		
Cliff’s $\delta$	$\delta$	0.272
	$P(X < Y)$	0.633
	*p*	0.048
Kruskal-Wallis	*p*	0.05
Wilcoxon Rank Sum	*p*	0.051

**Table 5:** Significance Test Results for **Response Time** – *Piggy Metrics*

Response Time (PM) - Control vs Exp.		
Cliff’s $\delta$	$\delta$	0.165
	$P(X < Y)$	0.594
	*p*	0.171
Kruskal-Wallis	*p*	0.241
Wilcoxon Rank Sum	*p*	0.243

Regarding the Response Time for Piggy Metrics, $\delta$ is again closer to zero. However, the $P(X<Y)$ value of 0.594 indicates at least that this time, the two curves do not overlap completely. Accordingly, we have a *p* value that is lower than we saw for the Response Time in the previous project but still high enough to not letting reject us our null hypothesis ${\textbf {H}}_{0,2}$ in this case.

According to our results, the difference in Correctness between the Control and Experiment Groups is significant for both projects. We thus reject our null hypothesis ${\textbf {H}}_{0,1}$ and accept ${\textbf {H}}_{A,1}$, which leads to the following conclusion regarding **RQ1**:

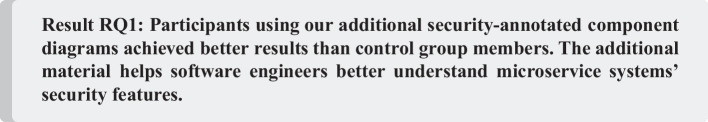



However, the same does not hold for Response Time: We found no significant difference, which let us reject our null hypothesis ${\textbf {H}}_{0,2}$. Therefore, we must assume our additional diagrams do not affect the participant’s response time.

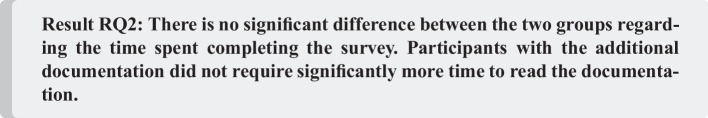



#### RQ3: Testing for Correlation

A common way to determine the relationship between two variables is to calculate Pearson’s correlation coefficient (Pearson 1895). Although frequently utilized, the requirements for its correct use are relatively strict. Again, one of its constraints is that the data must be normally distributed, a property which does not necessarily hold for our complete data set (see Section “Testing for Normal Distribution”). Instead, we use its non-parametric equivalent, Spearman’s rank correlation coefficient, also called Spearman’s $\rho$ (Spearman 1987), in combination with a visual confirmation through scatter plots. The results of our calculations for the *eShopOnContainers* and *Piggy Metrics* projects are shown in Tables 6 and 7.

**Table 6:** Correlation of Correctness and Response Time - *eShopOnContainers*

		Control	Experiment
Number of observations		34	36
Spearman’s	$\rho$	0.069	0.105
	*p*	0.72	0.569

**Fig. 4:**
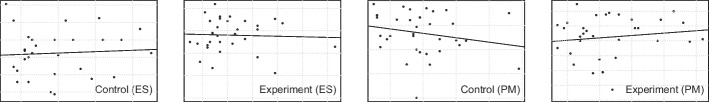
Scatter Plot for Response Time (x-axis, [min]) and Correctness (y-axis, [0;1]) *eShopOnContainers* (left) and *Piggy Metrics* (right)

The correlation coefficient $\rho$ expresses how strongly the two variables are related. The interpretation is often based on Cohen guidelines Cohen (2013), where values $\le 0.1$ are considered weak or have no correlation. According to this, both groups show none, respectively, only a very weak correlation. This observation is further supported by the significance measure *p*, which is both times higher than our $\alpha$ of 0.05, indicating that any found correlation would likely only be by chance. The scatter plots in Fig. 4 give additional insights: In the case of a linear correlation, most data points would lay directly or near the reference line, while in our diagram, the points are wildly scattered without any visible pattern.

Also, there seems to be a discrepancy between the negative slope in the *Experiment (ES)* scatter plot (Fig. 4, second from left) compared to the positive correlation coefficient 0.105 in Table 6. That happens because outliers, like the one visible in the bottom area of the scatter plot, have a relatively strong pull effect on the regression line, which, in our case, leads to a slightly negative slope. In contrast, the rank transformation applied when calculating Spearman’s $\rho$ reduces the impact of these outliers, resulting in divergent observations for the calculation and the diagram.

Testing the correlation of both variables for the *Piggy Metrics* gives us similar results: While the $\rho$ value of $-0.263$ for the Control Group is more pronounced than in the previous group, the *p* value is still relatively high, so again, a possible correlation is likely only by chance. Based on a $\rho$ closer to 0.1 and a considerably higher *p* value, the Experiment Group neither shows any correlation between Correctness and Response Time. Also, the scatter plots (Fig. 4) show no sign of a linear correlation. Meanwhile, at least some points are on or near the line for the Control Group, but most data points are too far away to allow any conclusion regarding the linear correlation.

Based on these results, we could neither identify any linear correlation between Correctness and Response Time for the Control nor the Experiment Group. Accordingly, we have to accept both null hypotheses ${\textbf {H}}_{0,3}$ and ${\textbf {H}}_{0,4}$ and reject their alternatives ${\textbf {H}}_{A,3}$ and ${\textbf {H}}_{A,4}$.

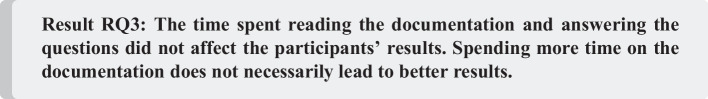



#### RQ4: Self-assessment Survey Results

In addition to the comprehension tasks, participants were also asked to answer a survey after finishing each project task. The questionnaire consisted of five-point symmetric Likert-Scale (Joshi et al. 2015) questions in descending order that asked about the candidate’s confidence when answering the tasks.

**Table 7:** Correlation of Correctness and Response Time - *Piggy Metrics*

		Control	Experiment
Number of observations		36	34
Spearman’s	$\rho$	-0.263	0.128
	*p*	0.127	0.477

In the first question, we asked the students to rate on a scale from one to five how much they thought their answers were right or wrong, where one expressed strong confidence that the answers were correct, while five points indicated no confidence at all.

We further combined their confidence rating with the actual Correctness value they achieved through solving a project’s tasks. Based on these two values, we calculated a self-assessment score $Assessment_{s}$ per student *s* to understand how strongly they underestimated or overestimated their performance. The score is calculated given the following formula:

$$ Assessment_{s} = Correctness_{s} - \frac{5 - Confidence_{s}}{5} $$

**Fig. 5:**
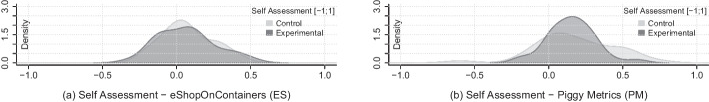
Kernel Density Plots for Self Assessment Score, *eShopOnContainers* (a) and *Piggy Metrics* (b)

An $Assessment_{s}$ score of 0 indicates that the participant estimated their performance accurately compared to their actual result. At the same time, values smaller than 0 suggest that their result was worse than they guessed. On the contrary, a score higher than 0 expresses an underestimation of their performance: Their results were better than they would have expected. The Tables 8 and 9, together with the kernel density plots in Fig. 5, summarize the $Assessment_{s}$ scores of the participants for each project, grouped by Control and Experiment Group.

**Table 8:** Descriptive Statistics for Self Assessment *eShopOnContainers*

	Control	Experiment
Number of observations	34	35
Mean	0.07	0.06
Median	0.05	0.09
Standard deviation	0.172	0.194
Minimum	-0.23	-0.32
Maximum	0.43	0.51
Skewness	0.274	0.371
Kurtosis	-0.762	-0.536

**Table 9:** Descriptive Statistics for Self Assessment *Piggy Metrics*

	Control	Experiment
Number of observations	36	34
Mean	0.18	0.15
Median	0.15	0.16
Standard deviation	0.265	0.159
Minimum	-0.6	-0.19
Maximum	0.73	0.6
Skewness	-0.253	0.272
Kurtosis	0.424	0.518

For the eShopOnContainers project, both groups assessed their performances relatively accurately. Both groups underestimated their performance for the Piggy Metrics project and achieved slightly better results than they thought. These results could also hint that the *Piggy Metrics* project was better accessible and maybe less complex than *eShopOnContainers*, an observation also seconded by the results we evaluated through our descriptive statistics analysis (see Section “Descriptive Statistics”).

**Fig. 6:**
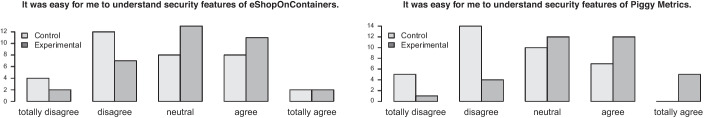
Frequency of answers to the question how easy it was to understand security features, ordered by group. Left: Results for *eShopOnContainers* project. Right: Results for *Piggy Metrics* project

We also asked participants to assess their comprehension of the systems’ security-related features. Figure 6 shows that for both Experiment Groups, we can observe a noticeable shift to the right regarding the self-assessed understanding of security tactics. While most Control Group members said understanding these features was somewhat challenging, the Experiment Group had a much better impression. However, only a few participants gave the highest score for this answer, so there still seems to be potential for supporting developers in this area.

**Fig. 7:**
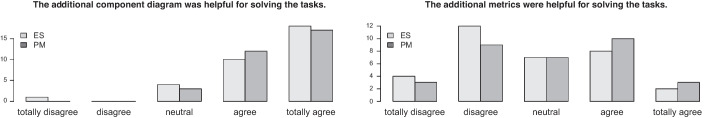
Results of how helpful the participants rated the additional component diagrams (left) and provided metrics (right)

Finally, we wanted to know how helpful our candidates would rate the additional tools, i.e., the component diagrams and the architecture metrics (see Sections 3.3 and 3.5). Here, only the answers from the Experiment Group are relevant, as these were the only ones with the additional tools. Consequently, the results are only grouped by the project this time (Fig. 7).

Concerning the component diagrams, a clear picture emerges: The vast majority found the charts extremely helpful across both projects, with *Piggy Metrics* having slightly higher approval rates.

However, the picture is divided regarding the metrics: For the *eShopOnContainers* project, most people found the metrics to be of little use. Depending on the interpretation, one could even assume they were counterproductive or caused more confusion than being helpful. While these are unexpected results, without further research, we can only speculate about a possible reason for disliking the metrics here. One reason might have been that, due to the manageable size of the projects, the visual representation provided by the diagrams was already sufficient, and the metrics were thus regarded as unnecessary.

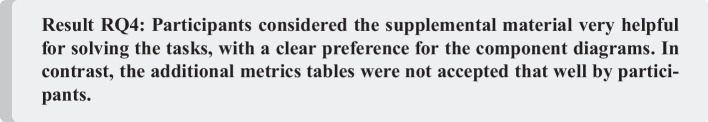



#### RQ5: Effect of Industry Experience on the Correctness

We also wanted to find out how strong a subject’s working expertise affects the results they achieve in the questionnaire. Intuitively, one would expect people with several years of industry experience to perform significantly better, at least in the Control Group (Tables 10 and 11).

**Fig. 8:**
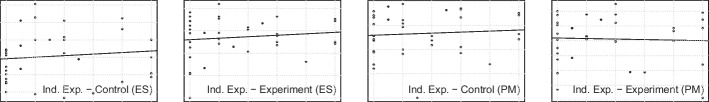
Scatter Plot for Industry Experience (x-axis, [years]) and Correctness (y-axis, [0;1]) *eShopOnContainers* (left) and *Piggy Metrics* (right)

Again, we calculated Spearman’s $\rho$ (Spearman 1987) to find a correlation between the two variables, a subject’s correctness, and their years of experience: While some coefficients indicate potential correlations, our *p* values are all above the $\alpha$ threshold. Also, a visual verification through the scatter plots (see Fig. 8) reveals no connection between the two variables. The dots are not concentrated around the reference line but instead are scattered around the area. Based on this observation, we can not postulate that a subject’s expertise does affect their test score.

While this may sound counterintuitive at first sight, there are several reasons for this conclusion: First, although most of our participants have industry experience, we only have a few subjects with five or more years of expertise. However, it could be possible that people need a certain level of experience until it affects the results significantly. When investigating the far right border of the scatter plots, we see more dots in the upper diagram area than at the bottom, but we do not have enough data to support this assumption, as these results could also be by chance. Additionally, industry experience is highly specialized within specific domains. For instance, a developer might have gathered multiple years of expertise in hardware or embedded systems without delving into distributed systems and their associated security considerations.

We also wanted to know whether our component diagrams have different effects based on a subject’s expertise, e.g., do novice developers benefit more from the diagrams than experienced developers? For this, we split our participants into three, almost equal-sized groups: *Novice* (no industry experience), *Junior* (up to two years of professional expertise), and *Senior* (three years and more), and investigated whether we could find any significant differences when comparing these groups. As dividing by expertise results in considerable small clusters, we decided not to divide further by separating into projects. While our groups remain relatively small (around 20 samples each), this should be enough to provide reliable results when using Cliff’s $\delta$.

**Fig. 9:**
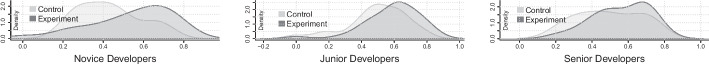
Kernel-Density plots for Correctness – *Control vs Experiment* grouped by expertise

Table 12 compares the significance coefficients between the Experiment and the Control Group, organized by expertise: Looking at novice and junior developers, the difference between both groups is recognizable due to larger $\delta$ values while having *p* values below our threshold ($\alpha = 0.05$). Also, in both cases, according to the $P(X<Y)$ values, the correctness results of the Experiment Groups are likely higher than in the Control Group, which supports our previous findings that the diagrams improve the correctness significantly. In contrast, the situation is unclear for senior developers: While $\delta$ is still high, the *p* value is also above our expected $\alpha$, indicating that the difference between the two groups is insignificant. Interestingly, the $\delta$ value shrinks from left to right, indicating that the difference between the Control and the Experiment Group reduces with increasing industry experience. The same effect can also be observed in the corresponding kernel-density plots (Fig. 9): While for the novice developers, a substantial distinction between Control and Experiment is visible, both curves get closer to each other for juniors and almost overlap for higher correctness values for senior developers.

**Table 10:** Correlation of Correctness and Industry Experience - *eShopOnContainers*

		Control	Experiment
Spearman’s	$\rho$	0.201	0.059
	*p*	0.255	0.731

From this observation, novice developers greatly benefit from the visual aid and structured representation provided by component diagrams, but experienced professionals exhibit a reduced dependency on these diagrams. Their knowledge enables them to compensate for the absence of these visual aids. They may also be more used to informal documentation due to their daily work and find extracting relevant information from textual descriptions easier.

Conversely, providing novice or inexperienced developers with additional component diagrams can significantly increase their understanding of security concepts and might even put them on the same level as much more seasoned developers.

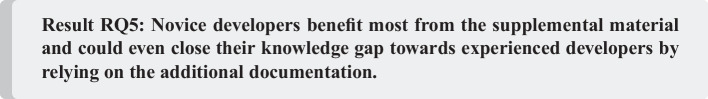



#### Qualitative Responses

Besides our statistical evaluation, we collected qualitative responses by talking to some participants directly after the survey. One aspect several individuals mentioned was the ambiguity of the information present in the informal textual documentation. Extracting the information relevant to answering the questions was sometimes challenging, and subjects often wondered whether their interpretation of the information was accurate. One expert also mentioned the importance of security in a distributed architecture. Thus, when in doubt, one must interpret the documentation pessimistically and assume that the given system needs to be more secure.

**Table 11:** Correlation of Correctness and Industry Experience - *Piggy Metrics*

		Control	Experiment
Spearman’s	$\rho$	0.116	0.044
	*p*	0.501	0.804

Others also mentioned that consulting the system’s source code instead of its textual documentation would generate much more certainty, as they consider the code the only honest source of truth regarding the actual architecture. With this in regard, our diagrams can also be seen as valid sources since we generate them directly from code with almost no room for interpretation – assuming that our generation process creates correct results. Nevertheless, repeating our experiment with the source code instead of textual documentation would be an exciting option for further research. However, this would require a different setup with even smaller systems, as reading through the whole code base of a sizeable microservice system is barely manageable within such a limited time frame.

When asked about the general structure of the questionnaire, some subjects complained that the questions that required a textual answer were too lengthy to answer. We even had one expert skip these questions due to timely limitations. Evaluating these questions can also be challenging, e.g., too much room for interpretation and the need to normalize the different spellings. Hence, we consider skipping these questions in future surveys to favor more accessible ones, like multiple-choice.

### Discussion and Interpretation of the Results

Our study shows that software developers achieved better results in assessing the security tactics used throughout a microservice system when provided with additional help through our security-annotated component diagrams. While both Control Groups achieved relatively low or average Correctness results (with a median of only 0.42 for the *eShopOnContainers* project), we recognized significant improvements in the Experiment Groups: For both projects, Experiment Group participants achieved an average of 0.16 higher Correctness values for the *eShopOnContainers* project and 0.12 for *Piggy Metrics*. Still, results for the *eShopOnContainers* project remain considerably low, even for the Experiment Group. This may be due to the project’s initial complexity and the partly ambiguous textual documentation. Although our additional diagrams could improve the situation, we assume that more sophisticated documentation would still be necessary to compensate for the project’s higher complexity.

**Table 12:** Significance Test Results for **Correctness** – *Control vs Experiment* grouped by expertise

Novice Developers		Junior Developers		Senior Developers	
Cliff’s $\delta$	0.375	Cliff’s $\delta$	0.316	Cliff’s $\delta$	0.215
$P(X < Y)$	0.663	$P(X < Y)$	0.648	$P(X < Y)$	0.614
*p*	0.046	*p*	0.044	*p*	0.192

For Response Time, we could not find any significant differences between the two groups. Both groups took nearly the same time to read the documentation and answer the questions. The *Piggy Metrics* Experiment Group took minimal longer to read the documentation. Still, the difference between the two groups was not significant. Conversely, this also means that the Experiment Group did not require more time to read the additional information, yet achieved better results than the Control Group. This may indicate that the component diagrams contain the relevant information more densely than the informal textual description, making this information more accessible. Our observations also showed that participants intuitively spent roughly a third of the time per project on the documentation and then went over to answer the tasks.

Overall, we consider this a very positive outcome: Using our security-annotated component diagrams improves the participants’ results without requiring more reading time. Even considering the implementation effort required to generate the diagrams, these costs will amortize soon. While the process requires some upfront implementation of the detectors for parsing the code base, these detectors are very lightweight and tailored to a specific project’s coding and technology guidelines, reducing the implementation time further (Zdun et al. 2023b). Also, once implemented, they can easily be reused - as long as the implementation does not introduce new technologies - and updated models can be generated with nearly zero costs.

Our analysis also revealed no correlation between Correctness and Response Time. Neither spending more nor less time reading the documentation and answering the tasks worsened or improved the results. We assume that, after a specific amount of time, the participants distilled all information they considered relevant from the underlying documentation, and spending additional time did not increase their knowledge of the system anymore.

Looking further at the self-assessment scores the participants gave themselves, most estimated their performance to be broadly accurate. Regarding the considerably lower Correctness values in the Control Group, this also means that the candidates were aware of the systems’ complexities and also recognized their potential knowledge deficits. Members of the *Piggy Metrics* Experiment Group marginally underestimated the Correctness of their results. Finding a concrete reason here is also difficult without further investigation. One possible explanation could be that they felt discouraged by the amount of information and, thus, rated the system’s complexity higher than it actually was.

Participants also agreed that the supplemental material helped them understand the systems’ security features better. However, while the diagrams were very well received, the opinions regarding the metrics were quite ambivalent. A possible reason could be that grasping both systems through diagrams was still manageable because of their limited service count. More complex architectures, on the other side, could have required much more sophisticated decomposition models, and gathering relevant information would then be easier by reading a single metric instead. However, further research is required to confirm this.

We also found that although years of experience did not automatically lead to better results, it helped reduce the difference between the Control and the Experiment Groups. While novice developers benefitted the most from the additional diagrams, participants who had already worked several years in the industry could compensate for the missing diagram information with their knowledge and experience.

## Threats to Validity

### Threats to Internal Validity

Eliminating the effects of confounding variables is inherently challenging, but we applied several measures to minimize their impact as much as possible: Each participant’s knowledge or experience is a factor that could highly intervene with the actual Correctness results in case individuals with higher knowledge distribute unevenly throughout the groups. Our within-subject design eliminates such asymmetric distribution of subject-level characteristics, as each individual was part of both groups. However, this setup can lead to learning or so-called carryover effects, where participants can reuse the knowledge they gathered during the first part of the experiment to solve the tasks of the second part. To countermeasure this effect, we randomized the order in which the different conditions, i.e., solving the tasks with or without the additional material, are presented to the participants, resulting in four different group combinations. On the downside, such a setup could lead to fatigue effects since participants now have to deal with two projects instead of one. While randomization also helps reduce this effect, we limited the experiment to 90 minutes and reduced the number of questions to keep the overall execution time as short as possible.

Regarding the online surveys with the industry experts, there is the risk that the participants used additional resources other than those provided, contrary to their assurances. While this may be possible, we consider this as relatively unlikely. Since we already provided parts of the documentation available for the given systems, it is doubtful they had found additional and highly qualitative resources helpful for solving our questionnaire. We would also consider the topic and the questionnaire too specific, as today’s AI systems like ChatGPT would be able to answer all of them correctly. However, the potential of these systems in assessing security architectures has yet to be better understood, and investigating this in more depth would undoubtedly provide opportunities for further research.

### Threats to External Validity

Although several industry experts took part in our survey, most participants were still students from different universities and not professional software developers. Therefore, transferring our results to the software industry must be done carefully. However, with two-thirds of our participants having industry experience, we would still argue that this is a solid basis for making our results relevant to the industry.

Another important consideration is whether the example projects we have selected represent today’s industry standards well. Albeit both projects being smaller than many real-world microservice systems, we consider them an adequate choice, as both are microservice reference implementations that use many best practices and technologies widely accepted by the industry. Both projects are also actively developed and can rely on a large community of experienced developers – *eShopOnContainers*, for instance, currently has more than 150 contributors and more than 24K stars on GitHub. While larger open-source microservice projects exist – like, for instance, the *Google Clouds Platform Demo*^18^ (or its derivative, *Hipstershop*^19^) or the *train-ticket* project^20^, the projects we chose are, because of their reduced size, easier to manage within the scope of a 90-minute controlled experiment. Analyzing larger projects would require more time and effort, resulting in potentially more substantial fatigue effects among the subjects. In addition, our projects’ documentation is less formal, which aligns well with the situation in real projects (Kleehaus and Matthes 2019).

Both systems are also quite mature and have been used in several other related research (see Section 4.2.5 for references), including studies with security-related contexts, like the one conducted by Nasab et al. (2021).

Nevertheless, repeating our experiments with a larger group of industry experts on commercial microservice architectures would be an exciting addition to our setup.

### Threats to Construct Validity

Our experiment focuses on how supplemental component diagrams and metrics can improve the understandability of security tactics used in microservice architectures. As such, our two dependent variables, Correctness and Response Time, are commonly used and provide well-suited indicators to measure the understandability of a subject (Paulweber et al. 2021; Czepa and Zdun 2018). The set of values (precision, recall, and f-measure) we used to calculate our Correctness is also relatively robust against potential cheating attempts.

Nevertheless, there may also be other viable ways than the ones we used to estimate the concept of understandability.

### Threats to Conclusion Validity

We missed some timestamps because participants forgot to record their time for specific tasks. While we were, in most cases, able to estimate the missing duration, we can not rule out that this may affect our statistical validity. The same applies to questions where we first had to transcribe the participants’ answers to bring them into a more standardized form for later processing. While unlikely, we may have misinterpreted single answers during this process.

We also took several measures to improve our statistical validity, like using a within-subject design experiment setup and applying statistical tests like Cliff’s Delta that work well for non-equally distributed data and smaller sample sizes. We also repeated the experiment in several rounds to ensure our sample count was large enough to achieve statistically relevant results.

### Relevance to Practice

We consider our results very relevant, as although widely adopted, microservice architectures can nevertheless be very complex systems, and understanding their security concepts is crucial for implementing them safely and robustly. This is even more important for systems running in the cloud as the chances of severe damage through malicious attacks or potential data leaks due to inadequate security measures are many times higher compared to running only in a local network (Miller et al. 2021).

Also, due to their rapid development and short iteration cycles, microservice documentation is often underdeveloped (de Toledo et al. 2019), making it harder for developers new to the team to catch up on all relevant security-related architectural decisions. Our findings suggest that having easy-to-understand documentation describing all relevant security aspects, combined with standardized diagrams, can help developers - particularly novices - better understand the systems, reducing the probability of implementing security-related bugs.

## Conclusion

In this study, we report on a controlled experiment to evaluate the efficiency of the security-annotated software composition models we generated as part of our previous work. These models are created by statically analyzing microservice code artifacts through lightweight source code detectors and are further enriched with architectural security tactics we collected from various literature research.

To determine whether our models and architectural metrics help software developers assess the security features of a microservice system, we conducted a within-subject-design experiment with 70 participants from two universities and the industry.

We estimated the effectiveness of the provided architectural artifacts by measuring the Correctness and Response Time of the participant’s answers to security-related questions on two open-source microservice reference implementations. Our results show that candidates provided with the supplemental diagrams and metrics achieved significantly higher Correctness scores when answering comprehension questions. We could also show that the benefit of our component diagrams decreases with the participants’ increased experience. Industry experts without additional diagrams could draw on their accumulated knowledge over the years to bridge the missing information. We also found that the additional material did not affect the Response Time required the total time for answering the questions. Despite consuming more material, the Experiment Group members took nearly the same time to read the documentation and answer questions. Besides this, we did not find any correlation between the Correctness of the answers and the time the participants spent solving the tasks, neither for the control nor the Experiment Group.

While participants appreciated the diagrams with security annotations, the additional architectural metrics were accepted more ambivalently. Some candidates found them helpful, but others saw little benefit. Nevertheless, further research will be required to compare the effects of visual diagrams and architectural metrics and to analyze how both could be combined more efficiently.

Our study highlights the usefulness of additional component diagrams for software and system documentation, especially in security aspects. These benefits can even be achieved with considerably low costs and resources, as our diagrams can, in large parts, be generated automatically.

## Figures and Tables

**Fig. 10:**
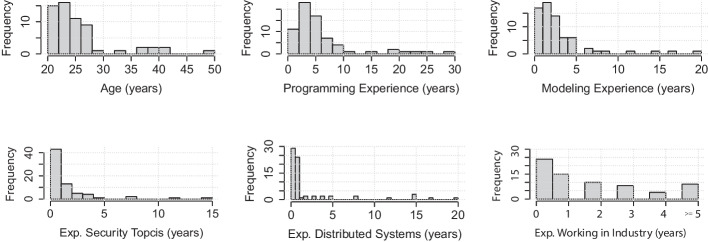
Distribution of Participants’ Demographical Data

**Fig. 11:**
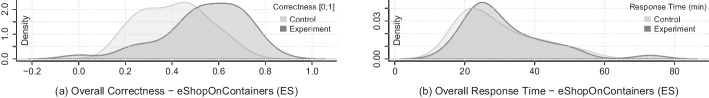
Kernel Density Plots for (a) Overall Correctness and (b) Total Response Time – *eShopOnContainers (ES)*

**Fig. 12:**
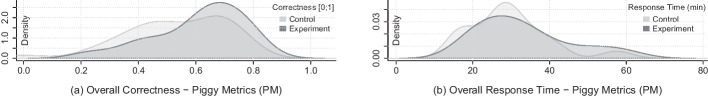
Kernel Density Plot for (a) Overall Correctness and (b) Total Response Time*Piggy Metrics (PM)*

**Fig. 13:**
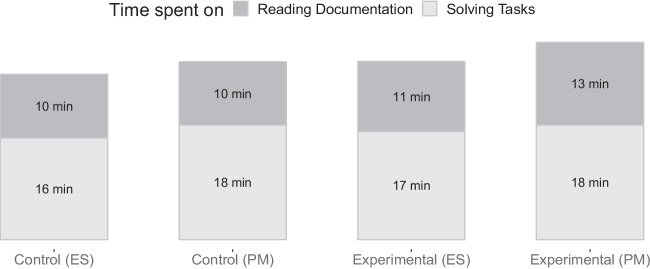
Time spent for reading the documentation and for solving tasks

**Fig. 14:**
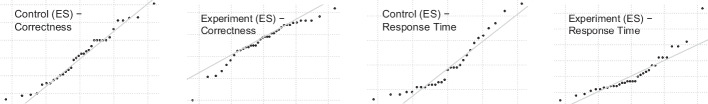
Q-Q Plots for Overall Correctness and Total Response Time – *eShopOnContainers (ES)*

**Fig. 15:**
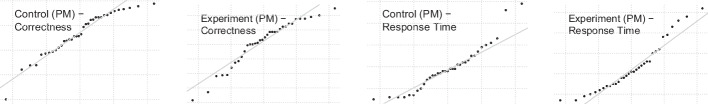
Q-Q Plots for Overall Correctness and Total Response Time – *Piggy Metrics (PM)*

## Data Availability

For supporting reproducibility, we offer all collected data of our study, together with the R-scripts used for analysis, and the questionnaires in a data set published on the long term archive Zenodo: https://zenodo.org/record/8089441.

## Appendix - Statistical Analysis

This section contains additional exploratory, demographics, and descriptive statistics of the dataset and concludes with a test for normal distribution. We performed our statistical analysis by utilizing the language R version 4^21^, in conjunction with the IDE RStudio 2022/2023^22^. We used the workflow and scripts similar to our previous experiment (Paulweber et al. 2021) but adapted them accordingly to our needs.

### Data-set Preparation

To measure the Correctness achieved when answering the survey, we computed the *precision* and *recall* values for each participant’s answers (De Lucia et al. 2010). The *precision* value determines how many of the answers a partipcant *p* gave were correct (De Lucia et al. 2010):

$$\begin{aligned} precision_p = \frac{\sum \limits _{i}\vert answer_{p,i} \cap correct_i \vert }{\sum \limits _{i}\vert answer_{p,i}\vert }\% \end{aligned}$$

The *recall* value expresses how many of the correct answers for a given task *i* the participant *p* found (De Lucia et al. 2010):

$$\begin{aligned} recall_p = \frac{\sum \limits _{i}\vert answer_{p,i} \cap correct_i \vert }{\sum \limits _{i}\vert correct_i\vert }\% \end{aligned}$$

Combing both measurements into a weighted *f-measure* score provides a more balanced result for the Correctness of each participant (De Lucia et al. 2010):

$$\begin{aligned} F-measure_p = 2 * \frac{precision_p * recall_p}{precision_p + recall_p}\% \end{aligned}$$

This approach is relatively robust against cheating in multiple-choice questions: Suppose a case where two out of five answers would be correct. A student could have checked all five options without reading the question and hoping to get a good result since they marked all correct answers. While this would result in a high *recall* value (all correct answers were given), the *precision* would be comparably low (as less than half of the given answers were correct).

The computed Correctness values were persisted into a new file for calculating the descriptive statistics.

**Table 13:** Descriptive Statistics for **Correctness** *eShopOnContainers*

	Control	Experiment
Number of observations	34	36
Mean	0.41	0.54
Median	0.42	0.58
Standard deviation	0.144	0.179
Minimum	0.17	0
Maximum	0.71	0.84
Skewness	0.127	-0.905
Kurtosis	-1.023	0.656

In case a candidate gave an inconclusive answer, e.g., by not filling out a blank, we rated this result as 0, meaning that not answering was equal to giving a wrong answer.

### Demographics

As part of our questionnaire, we gathered demographic and experience-related background information from the participants, including their age and gender, level of education (all optional), years of experience in programming and working with software-related diagrams like UML, and the number of years the participants have already worked professionally in the software industry. We also asked them to self-assess their years of experience in specific fields, such as security-relevant topics and distributed systems in general. Figure 10 shows the distributions of selected demographical data. Since the number of outliers was manageable for most responses, the median and the average differ only slightly, so we decided to use the median as our primary aggregator when describing the demographics.

Most students were between 20 and 25 years old, while the industry experts were mainly around 40, resulting in a median of *24* and a slightly higher average age of *26*. This distribution conforms with the educational level we collected from the participants (not visualized), as most of our students either had no academic degree yet (meaning they are currently doing their bachelor’s degree) or had their bachelor’s and are now doing their master’s. The third largest group was individuals with a master’s degree, mainly USI students and most industry experts.

When asked about their programming experience, most students stated it was between zero and five years. However, there were also individuals with more experience, reaching up to some of our industry experts having between 20 and 30 years of programming experience. Here, the median of *4.75* (mean: *6.34*) is not too far away from the aforementioned *Stack Overflow’s 2016 Survey* (see Section 4.2.3), where most developers had between two and five years, with the second and third largest cohort having more than five years.

As expected, modeling experience was, with a median of *2* years, lower than their programming experience – model-driven software development still has less widespread acceptance as part of software development (Petre 2013).

A similar distribution could be observed for experience in topics related to software security and general knowledge about distributed systems, where most participants either had almost no experience or reported their knowledge as very limited (both with a median of *1* year). It was also interesting to see that even our industry experts estimated their knowledge in this field relatively conservatively. However, one must also consider that these two terms can sometimes be quite expansive, and different participants may understand them differently. On the other hand, these numbers also indicate that security-related topics may not get the attention one would expect.

**Table 14:** Descriptive Statistics for Response Time *eShopOnContainers*

	Control	Experiment
Number of observations	29	32
Mean	29.55	31.34
Median	28	27
Standard deviation	10.891	12.122
Minimum	16	15
Maximum	55	73
Skewness	0.79	1.43
Kurtosis	-0.552	2.217

When asked about their practical experience working in the software industry, more than two-thirds of our participants answered they had worked at least one year professionally as software developers. The relatively large number of students with industry experience is typical for computer science studies, especially in Austria, as many start working in companies before finishing their studies. Compared to earlier semesters, the years of experience were even lower this time, most likely due to the preceding pandemic, which made it more difficult for students to find adequate jobs. With this large number of participants having work experience, we consider our audience a solid basis to make our results generalizable to the industry, at least to a certain extent. To keep the chart in Fig. 10 (bottom right) more readable, we grouped all senior levels ($\ge$ 5 years experience) into one bin.

**Table 15:** Descriptive Statistics for Correctness *Piggy Metrics*

	Control	Experiment
Number of observations	36	34
Mean	0.53	0.62
Median	0.52	0.64
Standard deviation	0.176	0.156
Minimum	0	0.21
Maximum	0.78	0.85
Skewness	-0.762	-0.788
Kurtosis	0.308	-0.179

**Table 16:** Descriptive Statistics for Response Time *Piggy Metrics*

	Control	Experiment
Number of observations	35	33
Mean	29.37	33.18
Median	28	31
Standard deviation	10.367	12.129
Minimum	15	17
Maximum	59	62
Skewness	0.907	0.775
Kurtosis	0.865	-0.393

### Descriptive Statistics

The goal of descriptive statistics is to summarize the characteristics of a given dataset by providing deeper insights into some statistical key indicators like the central tendency, variability, or distribution of the dataset. As we used two different systems – *eShopOnContainers* and *Piggy Metrics* – in our study, we investigate the results for each system separately.

#### eShopOnContainers

For the *eShopOnContainers*-related questionnaire, we had 34 samples for **Correctness** in the Control Group and 36 in the Experiment Group. In some rare cases, where participants did not answer a question or left it blank, we considered the corresponding questions as wrong answers and rated them with zero points. For example, in one such case, a candidate skipped the complete *eShopOnContainers* questionnaire, and we had to give zero points for their overall Correctness.

Table 13 summarizes the statistical values we collected for the tasks related to this project. The median and mean values were relatively close throughout our observation. According to these values, the Experiment Group also performed slightly better than the Control Group. Further, no individual in the Control Group reached a better result than in the Experiment Group. Although this could be interpreted as a first, early indication that supports our alternative hypothesis, additional tests were needed to validate whether these differences are statistically significant (see Section 4.4.1).

The last two measures (skewness and kurtosis) help to interpret the two groups’ distribution curve further: Skewness describes to what extent the curves are symmetrical – negative values, as in the case of Experiment, indicate that the distribution is left-skewed, i.e., having more values that are higher than in the case of a normal distribution. In contrast, Control’s skewness value is slightly above zero and thus more right-skewed. The sizeable negative kurtosis value of the Control Group signals that its distribution is platykurtic with more values around the center than in the edge areas, with the Experiment Group having its values more spread to the left (see also the Kernel Density plot in Fig. 11a for a visualization of these characteristics).

For the **Response Time**, we had to handle inconclusive values differently: Where possible, we estimated missing times by filling the gap between the times of the previous and upcoming tasks. However, this was not always applicable, as there were also questionnaires with more than a time record in a row missing. Therefore, we had to exclude several records where reliably reconstructing the Response Time was impossible.

Both distributions look close (see Table 14), with the Experiment Group having a more significant difference between its mean and median value, indicating that the values are less normally distributed here. Skewness values for both groups imply that both are left-skewed, suggesting that both groups answered the questions faster than expected from a normal distribution. Further, the Experiment Group has a much larger kurtosis value than the Control Group, indicating that more results are placed around the center (Fig. 11b).

#### Piggy metrics

For **Correctness**, the overall values for mean and median are higher, while the distance between the same groups stays almost the same. This may indicate that the project was more accessible to the participants than the *eShopOnContainers* (see Table 15 for more details). Although the skewness values of the Control and Experiment are within a similar range, the two curves look relatively different, with the Control curve being quite flat and widespread and the Experiment Group having more values around the center.

**Response Time** values are similar to the previous project. However, the higher mean and median values in the Experiment Group indicate that the candidates took more time to answer the question than for the previous project (see Table 16). Interestingly, the Control Group is more leptokurtic around the center, suggesting that candidates without the additional decomposition models needed less time to solve all tasks. In contrast, the Experiment Group is more platykurtic (Fig. 12b).

Comparing the time used for reading the documentation vs. the actual time spent on solving the tasks, the groups behave relatively homogenous, as illustrated by Fig. 13. One can say that participants spent roughly one-third of their time reading the documentation and the remaining two-thirds answering the questions, and this is throughout all groups. The *Piggy Metrics* Experiment Group is the only one where members spend more time on the documentation, which may explain why the Control Group had a smaller total Response Time.

**Table 17:** Normality Tests for Correctness *eShopOnContainers*

	Control	Experiment
Shapiro-Wilk *p*	0.4102	0.0476
Kolmogorov-Smirnov *p*	0.9318	0.7655
Anderson-Darling *p*	0.5065	0.0841

**Table 18:** Normality Tests for Response Time *eShopOnContainers*

	Control	Experiment
Shapiro-Wilk *p*	0.0098	0.001
Kolmogorov-Smirnov *p*	0.3194	0.1451
Anderson-Darling *p*	0.0078	0.0019

### Testing for Normal Distribution

Determining whether the difference of dependent variables between two groups is significant requires checking whether both populations are significantly different regarding their mean values.

As our statistical model consists of one independent variable (the membership of the individual to either the Control or the Experiment Group) and two dependent variables (Correctness and Response Time), a multivariant analysis of variance (MANOVA) would be a suitable method to test whether there is such a statistical significance between the variables and the group membership.

**Table 19:** Normality Tests for Correctness *Piggy Metrics*

	Control	Experiment
Shapiro-Wilk *p*	0.0379	0.0381
Kolmogorov-Smirnov *p*	0.6098	0.3546
Anderson-Darling *p*	0.1107	0.0379

**Table 20:** Normality Tests for Response Time *Piggy Metrics*

	Control	Experiment
Shapiro-Wilk *p*	0.0163	0.0223
Kolmogorov-Smirnov *p*	0.7505	0.5761
Anderson-Darling *p*	0.0762	0.0287

However, to apply a MANOVA, the data set must meet several criteria, the normal distribution of the data being one of them (French et al. 2008). Testing for normality can be done either with a formal, calculation-based approach or by analyzing the data visually. The kernel density plots we described earlier (Section “Descriptive Statistics”) can be used as an initial visual test: According to Hair Jr et al. (2021), skewness and kurtosis besides $-1/+1$ can indicate deviations from a normal distribution, which would especially be the case for some of our Response Times (see, for instance, Fig. 11b), but also for the Correctness values achieved by the ES Control Group (Fig. 11a). While this could be a first sign that our data may not be normally distributed, further analysis is needed to make a qualified decision.

We, therefore, applied an additional significance test to check our data set for normal distribution. According to various studies (Razali et al. 2011; Saculinggan and Balase 2013; Mendes and Pala 2003), the Shapiro-Wilk test (Shapiro and Wilk 1965) performs best for smaller sample sizes like the one used in our study and is superior to other tests like the Kolmogorov-Smirnov test (Kolmogorov 1933), and the Anderson-Darling test (Anderson and Darling 1954).

Tables 17-20 show the resulting *p* values of the Shapiro-Wilk tests, and for comparison, also the results of the two other tests mentioned.

Based on our outcomes, the response time results for both projects look normally distributed with $p < \alpha$ (see Tables 18 and 20). Also, the Correctness of the *PiggyMetrics* project shows a normal distribution for both groups (Table 19. However, the *p* values of *eShopOnContainers* Correctness groups look somewhat different: The Control Group’s *p* value 0.41 is way over the threshold, and the Experiment’s *p* value is borderline (Table 17). Accordingly, we may have at least one case with non-normally distributed data, if not even two.

We also considered Q-Q plots for visual verification as the last verification step. In the case of normally distributed data, the data points should be organized around the reference line, while dots far away from the line indicate less normally distributed data. While most of the dots of the Q-Q plots for the *eShopOnContainers* project (see Fig. 14) and the *PiggyMetrics* Response Time (Fig. 15) look centered around the line, the considerable large amount of outliers at the borders make a conclusive decision difficult, based only visual interpretation. The same applies to the Correctness of both projects, where we can see many outliers in the lower and upper areas of the chart. Based on this observation and some values of our Shapiro-Wilk test being over or at the threshold, we thus take a conservative approach here and assume our dataset is not normally distributed.

While authors like Norman (2010) argue that a MANOVA may be more robust against non-normally distribution than often assumed, Sarstedt et al. (2014) suggest that for using a parametric test like MANOVA, the sample size should at least consist of 30 observations. Since we barely reached this suggested number of observations per group and have valid concerns regarding the normal distribution of our data, we decided against a MANOVA in favor of using a non-parametric test procedure for validating our hypotheses.

## Footnotes

1 https://spec.openapis.org/oas/latest.html

2 https://www.asyncapi.com/docs/reference

3 https://github.com/sqshq/piggymetrics

4 https://www.oasis-open.org/committees/tc_home.php?wg_abbrev=security#overview

5 https://openid.net/connect/

6 https://jwt.io/introduction

7 https://github.com/dotnet-architecture/eShopOnContainers

8 https://github.com/sqshq/piggymetrics

9 https://www.usi.ch/

10 https://www.freecodecamp.org/news/2-out-of-3-developers-are-self-taught-and-other-insights-from-stack-overflow-s-2016-survey-of-50-8cf0ee5d4c21/

11 https://github.com/dotnet-architecture/eShopOnContainers

12 https://github.com/sqshq/piggymetrics

13 https://github.com/Netflix/zuul

14 https://github.com/Netflix/eureka

15 https://www.usi.ch/en/feeds/24840

16 https://www.google.com/forms/about/

17 https://rdrr.io/cran/orddom/man/orddom.html

18 https://github.com/GoogleCloudPlatform/microservices-demo

19 https://github.com/lightstep/hipster-shop

20 https://github.com/FudanSELab/train-ticket
